# Host–microbiota interaction drives 5‐hydroxyindole‐3‐acetic acid production to promote linear growth in infant mice

**DOI:** 10.1002/imo2.70062

**Published:** 2025-10-23

**Authors:** Yongmei Yang, Jing Yang, Weiyao Zhang, Linghao Zhou, Chenxu Zhu, Xuguang Zhang, Yuejian Mao, Meiling Zhang

**Affiliations:** ^1^ School of Life Sciences East China Normal University Shanghai China; ^2^ Global R&D Innovation Center Inner Mongolia Mengniu Dairy (Group) Co. Ltd. Inner Mongolia China

## Abstract

This study discovered that *Lactiplantibacillus plantarum* Hi188 promoted linear growth in postweaning mice. Transcriptomic analysis, untargeted metabolomics, and in vitro experiments showed that the elevated levels of 5‐hydroxyindole‐3‐acetic acid (5‐HIAA) activated the hepatic aryl hydrocarbon receptor (AhR) and subsequently promoted insulin‐like growth factor‐1 (IGF‐1) production via the IRS1/PI3K/Akt pathway. Integrated metagenomics and reverse transcription quantitative polymerase chain reaction (RT‐qPCR) analysis of host colonic genes revealed that 5‐HIAA was cooperatively produced by the gut microbiota and the host. These findings offered novel perspectives on the therapeutic potential of microbiota‐targeted interventions for growth regulation.

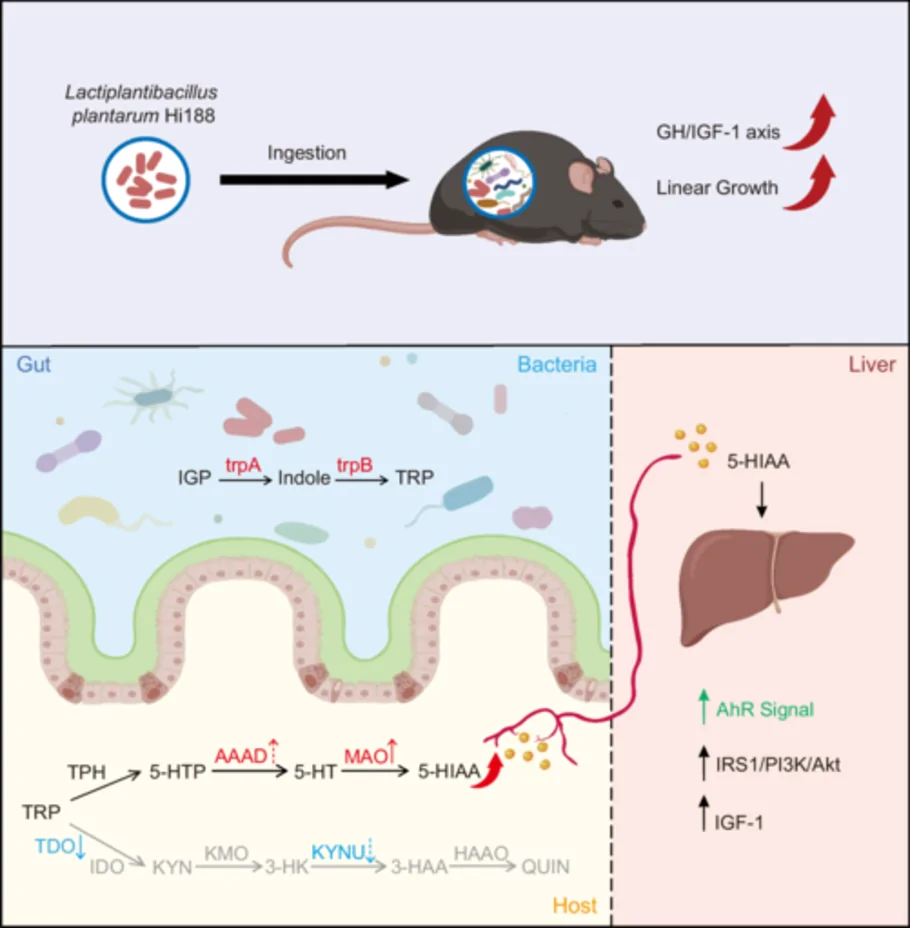

The gut microbiota plays a crucial role in human health, regulating host postnatal growth [1, 2], immune system development [3], nutrient absorption [4], and metabolic homeostasis maintenance [5]. Clinical observations have revealed that stunted children consistently exhibit characteristic gut microbial dysbiosis, marked by reduced microbial diversity [6], depletion of core functional taxa [7], and microbiota immaturity [8]. Notably, fecal microbial transplantation (FMT) from undernourished children to germ‐free (GF) mice successfully reproduces the growth stunting phenotype [8], establishing a causal link between gut microbiota and the growth impairment.

Accumulating evidence has demonstrated that probiotics effectively promote child growth and development [9]. Some research shows that *Lactobacillus rhamnosus* GG‐supplemented infant formula improves growth parameters versus standard formulations [10]. Multiple clinical trials show that probiotics improve weight and height gain in undernourished children, with potential benefits for well‐nourished children in developing countries [9]. The underlying mechanisms have also drawn widespread research interest. Compared to GF animals, mice colonized with conventional or specific *Lactobacillus* strains exhibit elevated insulin‐like growth factor‐1 (IGF‐1) levels and enhanced bone growth [2, 11]. *Muramyl dipeptide* (MDP), a peptidoglycan component of *Lactiplantibacillus plantarum* strain WJL, enhances the growth hormone sensitivity and circulating IGF‐1 levels through nucleotide‐binding oligomerization domain‐containing 2 (NOD2) receptor‐mediated interactions, consequently promoting growth in chronically malnourished mice [1]. It has been shown that probiotics can promote IGF‐1 expression by cell wall components, but whether probiotics have other mechanisms to promote growth remains unclear.

In the present study, we orally administered *L. plantarum* strain Hi188 to infant mice and assessed its effect on postweaning linear growth. We also investigated the mechanism by which *L. plantarum* Hi188‐induced growth promotion. Through untargeted serum metabolomic analysis and in vitro studies, we identified the critical metabolite 5‐HIAA and unveiled its potential in promoting growth. We elucidated a collaborative host‐microbiota mechanism for the biosynthesis of 5‐HIAA by integrating metagenomics and reverse transcription quantitative polymerase chain reaction (RT‐qPCR) analysis of host colonic genes. Our results suggested that gut microbiota collaborated with the host to coproduce 5‐HIAA, which stimulated IGF‐1 production, offering a potential microbiota‐targeted therapeutic strategy for promoting growth.

*L. plantarum* Hi188 was isolated from fermented tofu (Figure S1A). Whole‐genome sequencing revealed that this strain possessed 1 chromosome and 10 plasmids (Figure S1B). Growth characteristics and safety profile evaluations demonstrated that *L. plantarum* Hi188 was a safe probiotic candidate for further study (Figure S1C,D and Tables S1–S3).

To investigate the growth‐promoting efficacy of *L. plantarum* Hi188 in mice, male C57BL/6J mice after weaning were orally gavaged with sterile saline (CON group) or *L. plantarum* Hi188 (1 × 10^9^ CFU/day/mice, Hi188 group) from 3‐week‐old to 4‐week‐old, which was a rapid growth phase in mice (Figure 1A). After 1‐week of *L. plantarum* Hi188 gavage, mice exhibited significant linear growth in body length (Figure 1B–D) and bone (femur and tibia) length (Figure 1E,F). Hi188 group showed increased body weight (Figure 1G–I), while no significant effects were observed on organ weights (Figure 1J). Food intake did not differ significantly between the two groups (Figure 1K). Furthermore, *L. plantarum* Hi188 significantly increased small intestine length and showed an increasing trend in colon length (Figure 1L,M). These findings highlighted that *L. plantarum* Hi188 promoted postweaning growth in infant mice after 1 week of oral gavage.

**FIGURE 1:**
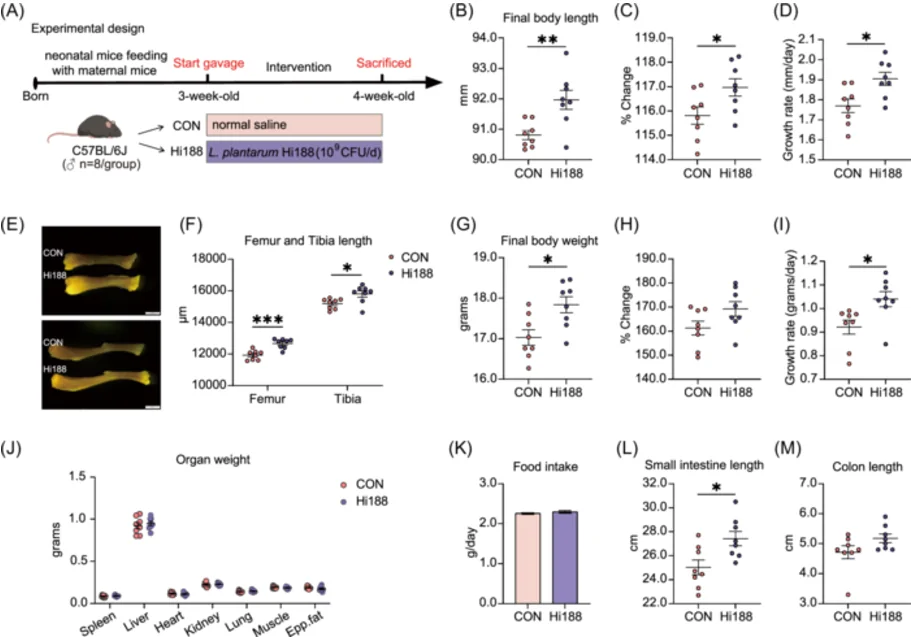
Growth phenotypes of mice gavaged with *L. plantarum* Hi188 for 1 week. (A) Experiment design. The male offspring were gavaged with saline (CON group), *L. plantarum* Hi188 (1 × 10^9^ CFU/day/mice, Hi188 group) from 3‐week‐old to 4‐week‐old, respectively (*n* = 8 per group). Final body length (B), relative body length change (C), and daily length increment (D) of 4‐week‐old mice in the CON and Hi188 groups. (E) Representative images of the femur (left) and tibia (right). Scale bars = 2 mm. (F) Quantification of femur and tibia bone length. Final body weight (G), relative body weight change (H), and daily weight gain (I) of 4‐week‐old mice in the CON and Hi188 groups. (J) Organ weight of 4‐week‐old mice. (K) Food intake of mice for 1 week. (L, M) Small intestine and colon length. Data were expressed as mean ± standard error of mean (SEM). Statistical significance was determined by unpaired two‐tailed Student's *t*‐test and denoted as *p < 0.05, **p < 0.01, and ***p < 0.001.

As the liver is a major site of IGF‐1 production, transcriptome analysis was performed on liver tissue to examine the growth‐promoting mechanism of *L. plantarum* Hi188. The Kyoto Encyclopedia of Genes and Genomes (KEGG) enrichment analysis revealed significant changes in growth‐related pathways between the CON and Hi188 groups, including insulin signaling pathway, insulin resistance, growth hormone synthesis, secretion, and action, along with the phosphatidylinositol 3‐kinase–protein kinase B (PI3K‐Akt) signaling pathway (Figure S2A). We found markedly increased serum IGF‐1 concentrations in the Hi188 group compared to the CON group (Figure S2B). Liver IGF‐1 levels showed an increasing trend (Figure S2C,D), while hepatic relative *Igf1* mRNA was significantly increased (Figure S2E). Relative growth hormone receptor (*Ghr*) mRNA expression also showed an increased trend in the Hi188 group, suggesting enhanced growth hormone (GH) sensitivity (Figure S2F). Further analysis showed increased insulin receptor substrate 1 (*Irs1*) mRNA expression (Figure S2G) and elevated phosphorylation levels of PI3K and Akt (Figure S2H,I), confirming IRS1/PI3K/Akt pathway activation in Hi188‐mediated growth promotion. These findings indicated that *L. plantarum* Hi188 promoted mouse growth through GH/IGF‐1 axis.

To identify the critical metabolite involved in host‐microbiota interactions that promoted the growth condition, untargeted metabolomic profiling of the mouse serum samples was performed by liquid chromatography‐mass spectrometry (LC‐MS) analysis. Principal component analysis (PCA) of differential metabolites showed distinct metabolic profiles in the CON and Hi188 groups (Figure S3A). KEGG pathway analysis showed that *L. plantarum* Hi188 gavage significantly altered serotonergic synapse, with concurrent modulation of tryptophan metabolism and phenylalanine/tyrosine/tryptophan biosynthesis (positive mode), as well as arachidonic acid metabolism (negative mode) (Figure 2A,B). Given that tryptophan metabolism may play a role in animal growth and IGF‐1 production [12], the relative abundances of all eight tryptophan‐related metabolites were compared between the two groups. Hi188 group exhibited a significant decrease in l‐tryptophan and indole, but a marked increase in 5‐HIAA (a downstream metabolite of serotonin) (Figure 2C and Figure S3B). Notably, 5‐HIAA was the singular upregulated metabolite among tryptophan‐related metabolites, and also a key component in the serotonergic synapse pathway, which regulates multiple growth‐related metabolic processes and forms a cross‐regulatory network connecting tryptophan and arachidonic acid metabolism [13]. While gut microbiota‐derived 5‐HIAA has been established to alleviate inflammation [14], improve metabolic disorders [15], and relieve diarrhea [16], we then sought to determine whether and how 5‐HIAA mediates the growth promotion.

**FIGURE 2:**
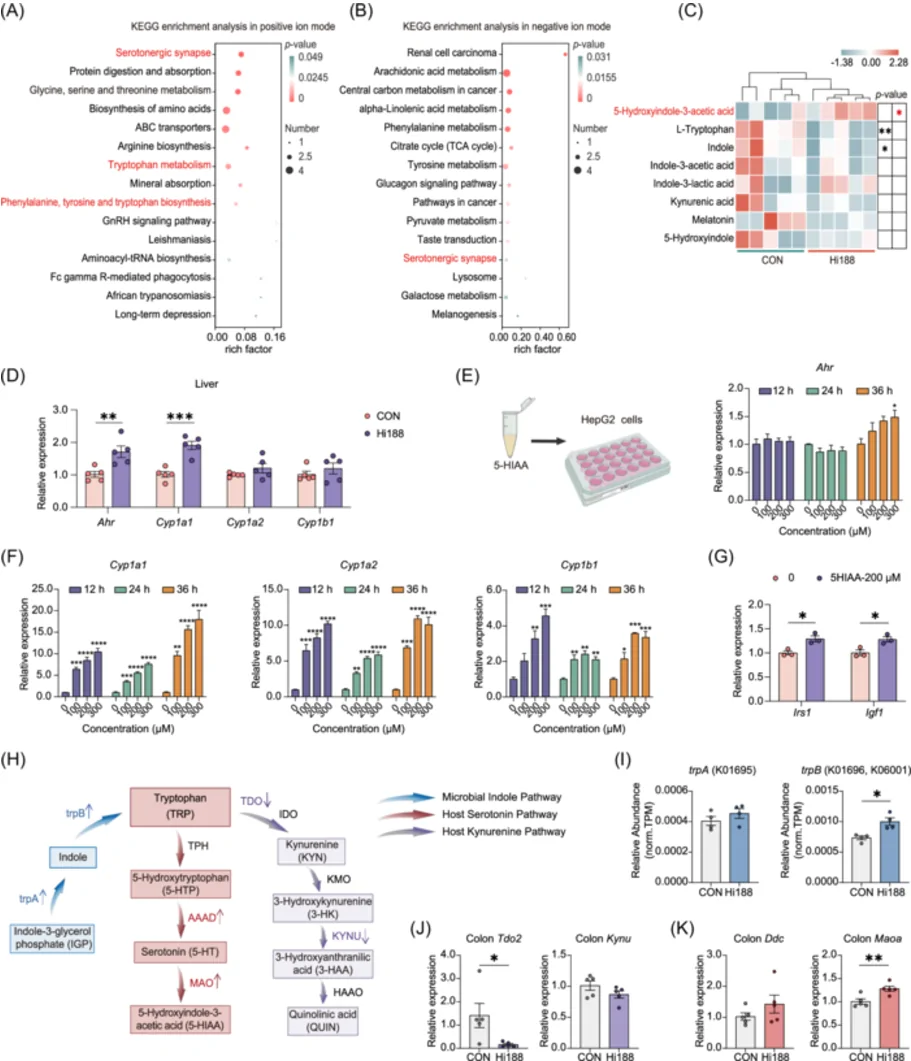
Gut microbiota and host enzymes coregulated 5‐hydroxyindole‐3‐acetic acid (5‐HIAA) production, stimulating growth via hepatic AhR/IRS1/IGF‐1 signaling in mice. (A, B) Untargeted metabolomic analysis of serum samples collected from CON and Hi188 groups (*n* = 5). Kyoto Encyclopedia of Genes and Genomes (KEGG) enrichment analysis of metabolites in positive and negative ion mode (Top 15 pathways ranked by *p*‐value). (C) The abundance of all tryptophan‐related metabolites in serum untargeted metabolomics between the CON and Hi188 groups. Red asterisks denoted significantly upregulated substances in the Hi188 group compared to the CON group; black asterisks denoted significantly downregulated substances. (D) Relative mRNA expression of aryl hydrocarbon receptor (*Ahr*), cytochrome P450 family 1 members (*Cyp1a1*, *Cyp1a2*, and *Cyp1b1*) in mouse liver tissue (*n* = 5). (E, F) Relative mRNA expression of *Ahr*, *Cyp1a1*, *Cyp1a2*, and *Cyp1b1* in HepG2 cells treated with 5‐HIAA (0, 100, 200, and 300 μM) for 12, 24, and 36 h (*n* = 3). (G) Relative mRNA expression of *Irs1* and *Igf1* in HepG2 cells treated with 200 μM 5‐HIAA for 36 h (*n* = 3). (H) Overview of the regulation pathways of tryptophan metabolism. Abbreviations: Tryptophan synthase subunit alpha (trpA), Tryptophan synthase subunit beta (trpB), Tryptophan hydroxylase (TPH), Aromatic l‐amino acid decarboxylase (AAAD; also known as Dopa decarboxylase, DDC), Monoamine oxidase (MAO), Tryptophan 2,3‐dioxygenase (TDO), Indoleamine 2,3‐dioxygenase (IDO), Kynurenine 3‐monooxygenase (KMO), Kynureninase (KYNU), 3‐Hydroxyanthranilate 3,4‐dioxygenase (HAAO). (I) Relative abundance of *trpA* (K01695) and *trpB* (K01696, K06001) associated with indole‐3‐glycerol phosphate (IGP) ‐to‐tryptophan conversion in the CON and Hi188 groups, data were obtained by metagenomic analysis (*n* = 4) (unpaired two‐tailed Student's *t*‐test, *p*‐value adjusted by the Benjamini–Hochberg method). (J) Relative mRNA expression of *Tdo2* and *Kynu* in mouse colon (*n* = 5). (K) Relative mRNA expression of *Ddc* and *Maoa* in mouse colon (*n* = 5). Data were expressed as mean ± SEM. The significant differences between the two groups were assessed based on unpaired two‐tailed Student's *t*‐test. One‐way ANOVA with Tukey's post hoc test was performed for multi‐group comparisons. Statistical significance was denoted as *p < 0.05, **p < 0.01, ***p < 0.001, and ****p < 0.0001.

Based on evidence that microbial indole derivatives activate AhR [5, 14] and the observed enrichment of AhR‐related pathways in our transcriptome analysis, we examined whether 5‐HIAA functions as an AhR ligand to stimulate hepatic AhR signaling. Hi188 treatment markedly upregulated the hepatic AhR and its primary marker gene, cytochrome P450 family 1 subfamily A member 1 (*Cyp1a1*) (Figure 2D)*. In vivo* findings suggested that elevated serum 5‐HIAA in the Hi188 group activated hepatic AhR, promoting IRS1/PI3K/Akt signaling and ultimately increasing both hepatic *Igf1* expression and serum IGF‐1 levels (Figure 2D and Figure S2A–I). The activation of the AhR signaling pathway was further verified by in vitro experiments in HepG2 cells. Cells were treated with 0, 100, 200, and 300 μM concentrations of 5‐HIAA for 12, 24, and 36 h. As expected, 5‐HIAA significantly activated AhR and upregulated its downstream marker genes (*Cyp1a1*, *Cyp1a2*, and *Cyp1b1*) (Figure 2E,F), thereby increasing the expression of *Irs1* and *Igf1* (Figure 2G).

To investigate the impact of gut microbiota on 5‐HIAA production, we conducted 16S rRNA sequencing of mouse cecal contents. Alpha(α)‐diversity indices had no significant differences between the CON and Hi188 groups (Figure S4A). Principal coordinate analysis (PCoA) and barplot analysis of the phylum level and the genus level revealed that *L. plantarum* Hi188 gavage induced certain compositional changes in the gut microbiota (Figure S4B–D). Random forest analysis identified the significantly enriched genus *Lactiplantibacillus* as the most significant discriminative feature (Figure S4E,F). Absolute quantification of fecal samples revealed that Hi188 group exhibited a significantly higher fecal abundance of species *Lactiplantibacillus plantarum* (Figure S4G). Importantly, *L. plantarum* Hi188 administration promoted the enrichment of 5‐HIAA‐producing microbial communities, including Burkholderiaceae_A [15], *Alistipes*_A [14], and *Ruminococcus*_B [17] (Figure S4H–J). Conversely, the Hi188 group exhibited the decreased abundance of 5‐HIAA‐inhibiting taxa, such as Peptostreptococcaceae [18] and *Turicibacter* [19] (Figure S4K,L).

To further investigate how 5‐HIAA was produced, we performed metagenomic analysis of cecal microbiota in these two groups using the Illumina NovaSeq platform. We found that the 5‐HIAA production was closely associated with tryptophan metabolism in both the host and gut microbiota. In the gastrointestinal tract, tryptophan metabolism primarily occurs through three main pathways [20] (Figure 2H). In the microbial indole pathways, metagenomic data indicated that the upregulation of genes *trpA* (encoding the α‐subunit of tryptophan synthase) and *trpB* (encoding the β‐subunit of tryptophan synthase) in the Hi188 group, facilitating the conversion of indole‐3‐glycerol phosphate (IGP) to tryptophan (Figure 2I). We also identified genes *trpA*, *trpB*, and *trpC* (encoding IGP synthase) in the genome of *L. plantarum* Hi188. These results indicated that the Hi188 group exhibited enhanced microbial tryptophan biosynthesis but attenuated indole metabolism. The consequent accumulation of tryptophan thereby served as a substrate for the downstream serotonin synthesis pathway.

Given that the kynurenine and serotonin pathways are host‐mediated and the colon is the key site for microbial‐host interactions [20], we measured colonic transcription of key enzyme genes in tryptophan metabolism. *L. plantarum* Hi188 gavage downregulated genes *Tdo2* (encoding tryptophan 2,3‐dioxygenase, TDO) and *Kynu* (encoding kynureninase) in the colon (Figure 2J), thereby inhibiting the kynurenine pathway and shifting tryptophan metabolism toward serotonin synthesis pathway. Kynurenine acid (Kyn) and its downstream metabolites, such as 3‐hydroxyanthranilic acid (3‐HAA), possess neurotoxic effects [20]. These changes further indicated a reduction in the accumulation of neurotoxic metabolites in the Hi188 group. Consequently, upregulated *Ddc* (encoding aromatic l‐amino acid decarboxylase, AAAD) and *Maoa* (encoding monoamine oxidase, MAO) helped to produce more 5‐HIAA (Figure 2K). These findings supported that serum 5‐HIAA elevation in the Hi188 group required the cooperation between gut microbiota and host tryptophan metabolic enzymes.

Our study unveiled a novel mechanism for stimulating postnatal growth in infant mice. Inspired by the work demonstrating that circulating 5‐HIAA activated hepatic AhR to enhance insulin signaling [15], we confirmed that 5‐HIAA activated hepatic AhR and subsequently stimulated IGF‐1 production. Further studies should employ exogenous 5‐HIAA supplementation and tissue‐specific AhR knockout models to reveal the underlying mechanisms.

Probiotics modulate the gut microbiota and metabolites, especially during the weaning period [12]. Our findings demonstrated that *L. plantarum* Hi188 exerted growth‐promoting effect in infant mice. Further analysis showed that *L. plantarum* Hi188 can facilitate asynergistic host‐microbiota crosstalk that enhanced tryptophan metabolism toward 5‐HIAA production. The elevated serum 5‐HIAA levels activated the AhR, triggering hepatic IGF‐1 synthesis, thereby stimulating GH/IGF‐1 axis and enhancing linear postweaning growth in infant mice. These findings clarified the growth‐promoting mechanism of *L. plantarum* Hi188, suggesting the potential of microbiome‐targeted interventions for host growth promotion.

## AUTHOR CONTRIBUTIONS

**Yongmei Yang**: Writing—original draft; investigation; methodology; formal analysis; conceptualization; software; validation. **Jing Yang**: Investigation. **Weiyao Zhang**: Validation. **Linghao Zhou**: Validation. **Chenxu Zhu**: Validation. **Xuguang Zhang**: Conceptualization. **Yuejian Mao**: Conceptualization. **Meiling Zhang**: Writing—review & editing; conceptualization; supervision; project administration. All authors have read the final manuscript and approved it for publication.

## CONFLICT OF INTEREST STATEMENT

The authors declare no conflicts of interest.

## ETHICS STATEMENT

All animal experiments conducted were approved by the East China Normal University (ECNU) Animal Care and Use Committee (Approval No. m20241003) and were in direct accordance with the Ministry of Science and Technology of the People's Republic of China on Animal Care guidelines.

## Supporting information


**Figure S1.** Characterization and safety assessment of *L*. *plantarum* Hi188.
**Figure S2.** *L*. *plantarum* Hi188 promoted mouse growth through GH/IGF‐1 axis.
**Figure S3.** *L*. *plantarum* Hi188 modulated the serum untargeted metabolome in mice.
**Figure S4.** *L*. *plantarum* Hi188 modulated gut microbiota in mice.

**Table S1.** Tolerance of artificial gastric juice, small intestinal fluid and bile salt of *L. plantarum* Hi188.
**Table S2.** Statistics of virulence factors in the *L. plantarum* Hi188 genome.
**Table S3.** Minimum inhibitory concentration (MIC) values and antibiotic resistance phenotypes for *L. plantarum* Hi188 and *L. plantarum* WCFS1.
**Table S4.** Primers used for quantitative real‐time PCR (qPCR).

## Data Availability

The raw data are available in the NCBI database under accession numbers PRJNA1293094 (liver transcriptome; https://www.ncbi.nlm.nih.gov/bioproject/PRJNA1293094), PRJNA1287344 (whole‐genome sequencing; https://www.ncbi.nlm.nih.gov/bioproject/PRJNA1287344), PRJNA1293133 (16S rRNA sequencing; https://www.ncbi.nlm.nih.gov/bioproject/PRJNA1293133), and PRJNA1293187 (metagenomic sequencing; https://www.ncbi.nlm.nih.gov/bioproject/PRJNA1293187). Metabolomics data have been deposited in the OMIX, China National Center for Bioinformation/Beijing Institute of Genomics, Chinese Academy of Sciences (accession number OMIX011083; https://ngdc.cncb.ac.cn/omix/release/OMIX011083). Supplementary materials (methods, figures, tables, graphical abstract, slides, videos, Chinese translated version, and update materials) may be found in the online DOI or iMetaOmics http://www.imeta.science/imetaomics/.
