# Supplementary material for: Host–microbiota interaction drives 5‐hydroxyindole‐3‐acetic acid production to promote linear growth in infant mice
Source: IMetaOmics. 2025 Oct 23;3(1):e70062. doi: 10.1002/imo2.70062 (PMC13674432; doi:10.1002/imo2.70062)
Supplement: Supplementary file 1 [file IMO2-3-e70062-s001.docx]

**Supporting information to**

**Host-microbiota interaction drives 5-hydroxyindole-3-acetic acid production to promote linear growth in infant mice**

**Running title:** 5‐HIAA promotes linear growth in infant mice

Yongmei Yang^1^, Jing Yang^2^, Weiyao Zhang^1^, Linghao Zhou^1^, Chenxu Zhu^1^, Xuguang Zhang^2^, Yuejian Mao^2*^, Meiling Zhang^1*^

^1^School of Life Sciences, East China Normal University, Shanghai 200241, China

^2^Global R&D Innovation Center, Inner Mongolia Mengniu Dairy (Group) Co. Ltd., Hohhot 011500, Inner Mongolia, China

^*^Correspondence: [mlzhang@bio.ecnu.edu.cn](mailto:mlzhang@bio.ecnu.edu.cn) (Meiling Zhang); [maoyuejian@mengniu.cn](mailto:maoyuejian@mengniu.cn) (Yuejian Mao)

**Methods**

**Bacteria information**

Lactiplantibacillus plantarum Hi188 was isolated from fermented tofu by our lab. *L. plantarum* WCFS1 (isolated from human oral saliva, ATCC BAA-793), used as the control strain for minimum inhibitory concentration (MIC) determination, was obtained from the laboratory of Baio Bowei Biotechnology Co., Ltd. (Wuhan, China).

Genomic DNA was extracted from *L. plantarum* Hi188, and 16S rRNA sequence was PCR-amplified using primer pair 27F (5’- AGAGTTTGATCMTGGCTCAG -3’) and 1492R (5’- GGTTACCTTGTTACGACTT -3’). The complete 16S rRNA gene sequence of *L. plantarum* Hi188 was available in NCBI with the GenBank number PP732472. A phylogenetic tree of the bacterial strains was constructed using MEGA v.7.0 software and then optimized using FigTree v1.4.3. Individual colonies of each bacterial strain were aseptically selected and transferred into the de Man, Rogosa and Sharpe (MRS) broth (BS1138, BaiSi Biotechnology, Hangzhou, China) and incubated at 37°C for 24–48 h before being used in subsequent experiments.

**Characterization and Safety Assessment of *L. plantarum* Hi188**

**Growth curve determination**

The growth of *L. plantarum* Hi188 was monitored during 28 h of continuous cultivation in MRS broth at 37°C. Single colonies were inoculated into 40 mL MRS broth at 200 rpm (37°C). At 2 h intervals, 200 μL of the homogenized bacterial suspension were collected, and the OD_600_ of bacteria was measured using the blank medium as the negative control. Three biological replicates were analyzed for each condition, and the mean optical density values were used to construct growth curves.

**Hemolytic test**

Single colonies of the isolated strain Hi188 were inoculated onto blood agar plates (OK3001, Oke Biotech, Anhui, China) at 37°C for 36 h. Then the hemolytic activity was assessed by observing the presence of clear zones around the colonies.

**Whole genome sequencing and information analysis**

*L. plantarum* Hi188 was cultured for 18 h at 37°C and then centrifuged at 12,000 × g (4 °C). The harvested cells were subsequently sent to Personal Biotechnology Co., Ltd. (Shanghai, China) for whole-genome sequencing. The bacterial genome was sequenced using Illumina NovaSeq and Oxford Nanopore Technologies (ONT) platforms. Third-generation sequencing data were assembled using Unicycler and Flye, corrected with Illumina reads via Pilon, and annotated with GeneMarkS for protein-coding gene prediction.

**Survival under conditions simulating the human gastrointestinal (GI) tract**

The strain's tolerance to the simulated human gastrointestinal (GI) tract was evaluated according to previously described methods [1–3]. *L. plantarum* Hi188 was cultured in 10 mL of MRS broth for 24 h. Bacterial cells (1 mL) were collected (3000 rpm, 5 min, 4°C), washed twice with sterile 1 × PBS (pH = 7.4) and resuspended in artificial gastric juice (pH = 3; PM20458, Canspec Scientific & Technology, Shanghai, China) or small intestinal fluid (pH = 6.8; PM20464, Canspec Scientific & Technology). The bacterial suspension was incubated at 200 rpm (37°C) for 3 h, with samples collected at 0, 1, 2, and 3 h for viable colony enumeration by plate counting.

The strain survival rates were calculated using the following formula: Survival rate (%) = (Nt/N₀) × 100, Nt represented the viable count at time t, and N₀ was the initial count at 0 h.

For bile salt testing, the test medium was prepared using MRS broth supplemented with 0.3% (w/v) bile salts (B8210, Solarbio, Beijing, China). The strain was cultured in MRS broth for 8 h. Bacterial cells were inoculated at 1% (v/v) into either: 40 mL of freshly prepared MRS broth (positive control), or broth medium containing 0.3% bile salts (treatment group). Each treatment and control group had three replicates. The bacterial suspensions were incubated at 200 rpm (37°C) for 3 h, with samples collected at 0, 1, 2, and 3 h for OD_600_ measurement.

Then the survival rates were calculated using the following formula: Survival rate (%) = (OD_treatment_ _group_ − OD_blank bile broth_) / (OD_positive_ _control_ − OD_blank MRS broth_) × 100

**Determination of minimum inhibitory concentration (MIC) values**

Seven antibiotics were tested in the broth microdilution method: ampicillin (60203ES10, Yeasen, Shanghai, China), gentamicin (S17024, Yuanye, Shanghai, China), kanamycin (60206ES10, Yeasen), erythromycin (E808820, Macklin, Shanghai, China), clindamycin (C168591, Aladdin, shanghai, China), tetracycline (S1705, Yuanye), and chloramphenicol (60205ES08, Yeasen). The procedure was conducted in accordance with the standard method outlined in ISO 10932/IDF 223 [4]. Bacterial resistance was evaluated based on the minimum inhibitory concentration (MIC). The tested concentration range was set at 0.5–256 μg/mL. Antibiotic stock solutions were prepared using the respective antibiotics. Each antibiotic was carefully weighed according to potency and dissolved in appropriate solvents, followed by thorough mixing. The medium and diluent used for MIC determination, was lactic acid bacteria susceptibility test medium (LSM) [5], which consisted of 90% Iso-Sensitest broth (IST; M8120C, TOPBIO, Shandong, China) and 10% MRS broth. The prepared high-concentration antibiotic stock solutions were diluted with LSM broth to twice the highest tested concentration.

The OD_600_ of each activated bacterial strain was measured, and cultures were diluted with LSM medium to achieve the desired density. A 1% inoculum was added to antibiotic dilution solutions at various concentrations, achieving a final inoculum density of 3 × 10^5^ CFU/mL. All suspensions were incubated in 96-well microplates at 37 °C. After 24 h culturing, the OD_600_ was measured. The lowest antibiotic concentration that inhibited bacterial growth was recorded as the MIC value. To validate assay reproducibility, the well-characterized strain *L. plantarum* WCFS1 was used as the control strain, with parallel testing of *L. plantarum* Hi188 to characterize its antibiotic resistance profile. Bacterial broth was used as the positive control (PC), while sterile broth served as the negative control (NC). All tests were performed in triplicate. In cases of inconsistent results among replicates, the experiment was repeated. The cut-off values were based on the European Food Safety Authority (EFSA) standard [6]. A bacterial strain was defined as resistant (R) when MIC > cut-off value, or susceptible (S) when MIC ≤ cut-off value.

**Experimental animals**

**Mice supplemented with *L. plantarum* Hi188**

The animal experiment was approved by the East China Normal University Institutional Animal Care and Use Committee (Approval No. m20241003). C57BL/6J mice, 8 to 9 weeks old and specific pathogen free (SPF), were obtained from ECNU Laboratory Animal Centre. Mice were mated, and male offspring were used for experiments. The male offspring were gavaged with saline (CON group), *L. plantarum* Hi188 (1 × 10^9^ CFU/day/mice, Hi188 group) from three-week-old to four-week-old, respectively (n = 8 per group). All mice were kept in the cages with a temperature range of 22–24℃, supplied with Co60-irradiated AIN-93G diet (XTM06-002, Synergetic Pharmaceutical Bioengineering Co., Ltd, Jiangsu, China) and water ad libitum, and maintained under a light-to-dark cycle of 12:12 h.

At the experimental endpoint, mice were fasted for 5 hours and then euthanized by isoflurane overdose followed by cervical dislocation. Blood samples were clotted at room temperature for 3–4 hours, centrifuged (3,000 rpm, 10 min, 4°C) and serum stored at -80°C until use. Tissues, including spleen, liver, heart, kidneys, lungs, gastrocnemius muscle and epididymal adipose tissue, were collected, weighed and immediately frozen. The small intestine and colon were measured for length before freezing, and cecal contents were carefully collected. All samples were maintained at -80°C until processing.

Before being fixed in 4% paraformaldehyde (PFA) solution, the left leg was photographed under a stereo microscope (OLYMPUS SZX16, Tokyo, Japan) and the lengths of the femur and tibia were measured.

**Serum and Liver IGF-1 measurement**

Serum insulin-like Growth Factor 1 (IGF-1) were detected by Mouse/Rat IGF-1 Quantikine ELISA (MG100, R&D systems, Minneapolis, MN) according to the manufacturer’s instructions. 20 mg liver tissue was homogenized in lysis buffer (mass: volume = 1:9) containing PBS and 1% phenylmethylsulfonyl fluoride (PMSF; ST507, Beyotime, Shanghai, China). After centrifugation (5,000 × g for 10 min, 4°C), the supernatant was collected, diluted 1:8 (v/v) and analyzed for IGF-1 levels following the kit protocol.

**RNA extraction and reverse transcription quantitative polymerase chain reaction (RT-qPCR)**

Total RNA was isolated from liver, colon and cell samples using Trizol Reagent (Invitrogen Life Technologies). After verifying integrity and quantifying by NanoDrop 2000, total RNA was reverse transcribed to complementary DNA using HiScript IV RT SuperMix (R423, Vazyme Biotech, Nanjing, China). The 2 × ChamQ Universal Qpcr SYBR Master Mix (Q771-02, Vazyme Biotech) was employed for quantitative real-time PCR (qPCR) analysis. *β-actin* was served as the reference gene to calculate target gene relative expression by 2^−ΔΔCT^ method. All primers sequences used in the study were listed in Table S4.

**Western blotting**

Liver tissue proteins were extracted using radioimmunoprecipitation assay (RIPA) buffer containing 1% PMSF at 4°C for 30 minutes. Protein samples were mixed with 5 × SDS loading buffer, boiled at 100°C for 10 minutes, then separated by 10% SDS-PAGE (P2012, New Cell & Molecular Biotech, Suzhou, China) and electro transferred to nitrocellulose (NC) membranes. Membrane blocking was carried out using QuickBlock™ Western blocking solution (P0252, Beyotime) for 20 minutes at room temperature. Primary antibody incubation was performed for approximately 14 hours at 4°C using the following specific antibodies: β-actin (66009-1-Ig, Proteintech, Wuhan, China; 1:5000 dilution); PI3 Kinase p85 alpha (T40116F, Abmart, Shanghai, China; 1:1000 dilution); Phospho-PI3-kinase p85- alpha/ gamma (Tyr467/199) (T40115F, Abmart; 1:1000 dilution); Akt (T55561F, Abmart; 1:1000 dilution); Phospho-Akt (Ser473) (T40067F, Abmart; 1:1000 dilution). Subsequently, membranes were incubated with secondary antibodies HRP goat anti-rabbit IgG (BF03008, Biodragon Technology Co., Ltd., Suzhou, China), HRP goat anti-mouse IgG (BF03001, Biodragon Technology Co., Ltd.) for 1 h and finally visualized using Chemidoc mp imager (Chemidoc mp, BIORAD, Hercules, CA).

**Transcriptome analysis of liver**

Total RNA was isolated from the liver (five samples per group), assessed for quality, and the purified RNA was used for library preparation with the Illumina Stranded mRNA Prep Ligation Kit (20040534, Illumina, San Diego, CA). The library was quantified using a Qubit 4.0 Fluorometer and sequenced on the Illumina NovaSeq X Plus platform (PE150) by Majorbio Bio-Pharm Technology Co., Ltd (Shanghai, China). After quality control, the clean data for each sample exceeded 5.93 GB and was aligned to the *Mus musculus* reference genome GRCm39 (GCA_000001635.9) using HISAT2 and assembled by String Tie. Differential expression analysis between the CON and Hi188 groups was performed using the DEGSeq2, with screening thresholds set at a *p*-value < 0.05 and fold change ≥ 1.5. Functional enrichment analysis of DEGs was conducted using the SciPy library (v1.11.0) in Python to identify significantly enriched KEGG pathways (Fisher’s exact test, *p*-value < 0.05). These pathways were further analyzed to elucidate the biological functions associated with the observed transcriptional changes.

**Untargeted serum metabolomics assay**

The serum samples were sent to Personal Biotechnology Co., Ltd. for untargeted metabolomics analysis by liquid chromatograph mass spectrometer (LC‐MS). Principal component analysis (PCA) was conducted by the R package Ropls for dimensionality reduction on sample data. Differential metabolites were hierarchically clustered and visualized as a heatmap using Pheatmap package (v1.0.12). Functional annotation was conducted through KEGG pathway enrichment analysis with clusterProfiler (v4.6.0), identifying statistically significant metabolic pathways.

**16S rRNA gene sequencing and data analysis**

High-quality DNA was extracted from cecal contents (five samples per group) using the MagBeads FastDNA Kit for Soil (116564384, MP Biomedicals, Solon, OH) for 16S rRNA sequencing and subsequent metagenomic analysis. The bacterial 16S rDNA (V3-V4 region) was amplified using pair primers 338F (5’- ACTCCTACGGGAGGCAGCA -3’) and 806R (5’- GGACTACHVGGGTWTCTAAT -3’). The PCR products were then purified, quantified, and sequenced on the Illumina NovaSeq platform (Personal Biotechnology Co., Ltd.).

Microbiome bioinformatics analysis was conducted using QIIME2 (v2024.5). Taxonomic composition and relative abundance were visualized with MEGAN and GraPhlAn. Alpha diversity was assessed through Chao1, Faith's Phylogenetic Diversity, observed features (Goods coverage), Shannon, and Simpson indices, calculated in QIIME2. Beta diversity was evaluated based on Bray–Curtis dissimilarity and visualized using principal coordinate analysis (PCoA). For predictive modeling, random forest analysis was implemented in R (v4.3.3) via the mlr3 package's classif.ranger() function (v0.22.0). Differential species abundance between the CON and Hi188 groups was determined using Mann–Whitney *U* test.

**Metagenomic analysis**

DNA libraries for shotgun metagenomic sequencing were prepared with 400 bp inserts utilizing the Illumina TruSeq Nano DNA LT Library Prep Kit. Paired-end sequencing (2 × 150 bp) was subsequently performed on an Illumina NovaSeq system at Personal Biotechnology Co., Ltd. The metagenomic sequencing generated an average of 10.36 ± 0.30 Gb raw reads per sample, with 10.11 ± 0.31 Gb (97.57%) clean reads retained after quality control. Sequences were aligned to the GRCm39 mouse reference genome (GCA_000001635.9) followed by host-DNA depletion using minimap2 (v2.24). Microbial reads were *de novo* assembled into contigs with MEGAHIT (v1.2.9), representing 77.9 ± 10.0% of total reads.

Open reading frames (ORFs) and coding regions were predicted from the assembled contigs using Prodigal. Gene and protein sequences were subsequently clustered and dereplicated using the MMseqs2 cluster module. Gene abundance in metagenomic data was normalized using TPM (Transcripts Per Million). Differential gene abundance between the CON and Hi188 groups was assessed using unpaired two-tailed Student’s *t*-test, with significance determined by a Benjamini–Hochberg adjusted *p*-value of < 0.05. Upon obtaining quality-filtered reads, taxonomic classification of each sample was executed using Kraken2 against a customized database (containing nucleotide sequences of bacteria and archaea from GTDB, eukaryotic microorganisms from NCBI-nt, and viruses from RVDB).

**Quantification of *L. plantarum* in fecal samples**

The target *L. plantarum* fragment was PCR-amplified, gel-purified (9762, TaKaRa, Beijing, China), and cloned into pMD19-T vector (6013, TaKaRa) for transformation into *Escherichia coli* BL21 competent cells (11804ES, Yeasen). Positive clones were selected by blue-white screening, and recombinant plasmids were verified by plasmid extraction and PCR before being commercially sequenced by Personal Biotechnology Co., Ltd. The recombinant plasmid carrying the *L. plantarum* V3-V4 region was quantified, and its copy number (copies/mL) was obtained using the formula: plasmid copies/mL = [6.02 × 10²³ × plasmid concentration (g/mL)] / plasmid molecular weight (g/mol). The plasmid was then serially diluted to generate seven consecutive 10-fold dilution gradients (10²–10⁸ copies/μL), with each concentration prepared in triplicate. qPCR was then performed to construct a standard curve for absolute quantification.

Quantification of *L. plantarum* in fecal DNA was performed by qPCR with primers specific to *Lactiplantibacillus plantarum* (Table S4) [7]. Genomic DNA was isolated from fecal samples of mice subjected to continuous 3-day gavage with Hi188, using the TIANamp Bacteria DNA Kit (DP302-02, Tiangen Biotech, Beijing, China).

**Cell culture and treatment**

The human hepatocellular carcinoma cell line HepG2 was obtained from QuiCell Biotechnology (H086, Shanghai, China) and maintained in complete growth medium (GM; TCH-G196, Haixing Biosciences, Suzhou, China). With reference to the dosage used in previous literature [8,9], 5-Hydroxyindole-3-acetic acid (5-HIAA; HY-W008253, MedChemExpress, Monmouth Junction, NJ) was dissolved in DMSO and diluted in GM to final concentrations of 0, 100, 200, and 300 μM. HepG2 cells were treated with 5-HIAA for 12, 24, and 36 h. All cells were cultured at 37°C in a humidified 5% CO₂ atmosphere and confirmed mycoplasma-free.

**Statistical analysis**

The data was performed as mean ± standard error of the mean (SEM). Normality and homogeneity of variance were assessed using Shapiro-Wilk and Levene's tests, respectively. The growth phenotypes, gene expressions, IGF-1 levels, metabolite levels were analyzed by unpaired two-tailed Student’s *t*-test or one-way ANOVA with Tukey's post hoc test. The 16S rRNA sequencing data were assessed using the Mann–Whitney *U* test. Statistical analyses were conducted using GraphPad Prism 10.1.0 and R (version 4.3.3), with data visualization assisted by an online platform (https://www.genescloud.cn). All reported *p*-values were two-sided. Significance levels were denoted as follows: **p* < 0.05, ***p* < 0.01, ****p* < 0.001, and *****p* < 0.0001.

**Supplementary figures**

**Figure S1** **Characterization and safety assessment of *L. plantarum* Hi188.** (A) The Neighbor-Joining phylogenetic tree of *L. plantarum* Hi188 based on 16S rRNA genes. The numbers above branches represented bootstrap support values (based on 1000 replicates). The scale bar indicated 0.02 substitutions per site. (B) The chromosome genome map of *L. plantarum* Hi188. The innermost circle represented the GC skew (Circle 1) in purple/green and GC content (Circle 2) in black. Circles 3 and 4 displayed the genomic features of *L. plantarum* Hi188, including protein-coding genes (PCGs, blue arrows), transfer RNAs (tRNAs, red arrows), and ribosomal RNAs (rRNAs, purple arrows), annotated on the majority (J-strand) and minority (N-strand) strands, respectively. (C) The growth curve of *L. plantarum* Hi188 (n = 3). (D) The colonies of *L. plantarum* Hi188 on blood agar.

**Figure S2 *L. plantarum* Hi188 promoted mouse growth through GH/IGF-1 axis.** (A) The differential KEGG enrichment analysis between the CON and Hi188 groups (n = 5). (B–D) The concentration of IGF-1 in serum and liver (n = 5). (E–G) Relative mRNA expression of insulin-like growth factor 1 (*Igf1*), growth hormone receptor (*Ghr*) and insulin receptor substrate 1 (*Irs1*) expression in mouse liver (n = 5). (H) The protein expression of phosphorylated phosphatidylinositol 3-kinase (p-PI3K) and total PI3K (n = 3). (I) The protein expression of phosphorylated protein kinase B (p-Akt) and total Akt (n = 3). Data were expressed as mean ± SEM. Statistical significance was determined by unpaired two-tailed Student’s *t*-test and denoted as ***p* < 0.01.

**Figure S3** ***L. plantarum* Hi188 modulated the serum untargeted metabolome in mice.** (A) Principal component analysis of mouse serum metabolic profiles. (B) The response intensity of all tryptophan-related metabolites in serum untargeted metabolomics between the CON and Hi188 groups (n = 5). Data were expressed as mean ± SEM. Statistical significance was determined by unpaired two-tailed Student’s *t*-test and denoted as **p* < 0.05, and ***p* < 0.01.

**Figure S4 *L. plantarum* Hi188 modulated gut microbiota in mice.** (A) Alpha diversity of gut microbiota was assessed using the Chao1, Shannon, Simpson, Goods coverage, Observed species (Mann–Whitney *U* test). (B) Principal coordinates analysis (PCoA) of gut microbiota based on Bray–Curtis dissimilarities. (C–D) Barplot analysis of the phylum level and the genus level of the gut microbial community. (E) Random forest analysis for genus differences and biomarker discovery between the CON and Hi188 Groups. (F) Relative abundance of genus *Lactiplantibacillus* (n = 5) (Mann–Whitney *U* test). (G) Absolute abundance of *L. plantarum* in fecal samples from mice after 3-day continuous gavage with Hi188 (n = 5) (unpaired two-tailed Student’s *t*-test). (H–J) Relative abundance of family Burkholderiaceae_A, genus *Alistipes_A* and genus *Ruminococcus_B* (n = 5) (Mann–Whitney *U* test). (K–L) Relative abundance of family Peptostreptococcaceae and genus *Turicibacter* (n = 5) (Mann–Whitney *U* test). Data were expressed as mean ± SEM. Statistical significance was denoted as **p* < 0.05, ****p* < 0.001, and *****p* < 0.0001.

**References**

1. Guo, Zhuang, Jicheng Wang, Liya Yan, Wei Chen, Xiaoming Liu, Heping Zhang. 2009. “*In Vitro* Comparison of Probiotic Properties of *Lactobacillus casei* Zhang, a Potential New Probiotic, with Selected Probiotic Strains.” *LWT-Food Science and Technology* 42: 1640–1646. <https://doi.org/10.1016/j.lwt.2009.05.025>

2. Xiong, Jie, Lingxing Feng, Muhammad Yousaf, Suping Zeng, Jun Tang, Yaping Wu, Qinqin Li, Dongmei Liu. 2024. “Assessment of Uric Acid-Lowering Activity, Safety and Stress Tolerance of *Lactiplantibacillus plantarum* MC14 Based on Whole Gene Sequencing and Phenotyping Experiments.” *Food Bioscience* 61: 104536. <https://doi.org/10.1016/j.fbio.2024.104536>

3. Maragkoudakis, Petros A, Georgia Zoumpopoulou, Christos Miaris, George Kalantzopoulos, Bruno Pot, Effie Tsakalidou. 2006. “Probiotic Potential of *Lactobacillus* Strains Isolated from Dairy Products.” *International Dairy Journal* 16: 189–199. <https://doi.org/10.1016/j.idairyj.2005.02.009>

4. International Organization for Standardization. 2010. “Milk and milk products—Determination of the minimal inhibitory concentration (MIC) of antibiotics applicable to bifidobacteria and non-enterococcal lactic acid bacteria (LAB) (ISO 10932:2010; IDF 223:2010). ” *International Organisation for Standardisation*, Geneva, Switzerland.

5. Klare, Ingo, Carola Konstabel, Sibylle Müller-Bertling, Rolf Reissbrodt, Geert Huys, Marc Vancanneyt, Jean Swings, Herman Goossens, Wolfgang Witte. 2005. “Evaluation of New Broth Media for Microdilution Antibiotic Susceptibility Testing of Lactobacilli, Pediococci, Lactococci, and Bifidobacteria.” *Applied and Environmental Microbiology* 71: 8982–8986. <https://doi.org/10.1128/AEM.71.12.8982-8986.2005>

6. EFSA Panel on Biological Hazards (BIOHAZ), Antonia Ricci, Ana Allende, Declan Bolton, Marianne Chemaly, Robert Davies, Rosina Girones, et al. 2018. “Statement on the Update of the List of QPS-Recommended Biological Agents Intentionally Added to Food or Feed as Notified to EFSA 7: Suitability of Taxonomic Units Notified to EFSA until September 2017.” *EFSA Journal* 16: 5131, 5143 pp. <https://doi.org/10.2903/j.efsa.2018.5131>

7. Choi, Chang Hun, Eiseul Kim, Seung Min Yang, Da Som Kim, Seung Man Suh, Ga Young Lee, Hae Yeong Kim. 2022. “Comparison of Real-Time PCR and Droplet Digital PCR for the Quantitative Detection of *Lactiplantibacillus plantarum* subsp. *plantarum*.” *Foods* 11: 1331. <https://doi.org/10.3390/foods11091331>

8. Du, Wei, Shanshan Jiang, Shengxiang Yin, Rongjiang Wang, Chunling Zhang, BinCheng Yin, Jialin Li, et al. 2024. “The Microbiota-Dependent Tryptophan Metabolite Alleviates High-Fat Diet–Induced Insulin Resistance through the Hepatic AhR/TSC2/mTORC1 Axis.” *Proceedings of the National Academy of Sciences* 121: e2400385121. <https://doi.org/10.1073/pnas.2400385121>

9. Zhong, Shi, YuQing Sun, JinXi Huo, WenYi Xu, YaNan Yang, Junbo Yang, WeiJie Wu, YongXin Liu, Chongming Wu, YouGui Li. 2024. “The Gut Microbiota-Aromatic Hydrocarbon Receptor (AhR) Axis Mediates the Anticolitic Effect of Polyphenol-Rich Extracts from *Sanghuangporus*.” *iMeta* 3: e180. <https://doi.org/10.1002/imt2.180>
